# Long-range replica exchange molecular dynamics guided drug repurposing against tyrosine kinase PtkA of *Mycobacterium tuberculosis*

**DOI:** 10.1038/s41598-020-61132-w

**Published:** 2020-03-10

**Authors:** Priya Nagpal, Salma Jamal, Hina Singh, Waseem Ali, Sana Tanweer, Rahul Sharma, Abhinav Grover, Sonam Grover

**Affiliations:** 1 School of Biotechnology, Jawaharlal Nehru University, New Delhi, 110067 India; 2 Jamia Hamdard Institute of Molecular Medicine, Jamia Hamdard, New Delhi, 110062 India

**Keywords:** Computational biology and bioinformatics, Virtual drug screening

## Abstract

Tuberculosis (TB) is a leading cause of death worldwide and its impact has intensified due to the emergence of multi drug-resistant (MDR) and extensively drug-resistant (XDR) TB strains. Protein phosphorylation plays a vital role in the virulence of *Mycobacterium tuberculosis* (*M.tb*) mediated by protein kinases. Protein tyrosine phosphatase A (MptpA) undergoes phosphorylation by a unique tyrosine-specific kinase, protein tyrosine kinase A (PtkA), identified in the *M.tb* genome. PtkA phosphorylates PtpA on the tyrosine residues at positions 128 and 129, thereby increasing PtpA activity and promoting pathogenicity of MptpA. In the present study, we performed an extensive investigation of the conformational behavior of the intrinsically disordered domain (IDD) of PtkA using replica exchange molecular dynamics simulations. Long-term molecular dynamics (MD) simulations were performed to elucidate the role of IDD on the catalytic activity of kinase core domain (KCD) of PtkA. This was followed by identification of the probable inhibitors of PtkA using drug repurposing to block the PtpA-PtkA interaction. The inhibitory role of IDD on KCD has already been established; however, various analyses conducted in the present study showed that IDD_PtkA_ had a greater inhibitory effect on the catalytic activity of KCD_PtkA_ in the presence of the drugs esculin and inosine pranobex. The binding of drugs to PtkA resulted in formation of stable complexes, indicating that these two drugs are potentially useful as inhibitors of *M.tb*.

## Introduction

*Mycobacterium tuberculosis* (*M.tb*) is an infectious agent that causes tuberculosis (TB) which is an airborne disease that affects almost one third of the world population. The Global Tuberculosis Report (2018), published by World Health Organization, estimated 1.3 million deaths and around 10 million new cases due to TB^1^. The currently available treatments for TB include first-line drugs (isoniazid, rifampicin, ethambutol, and pyrazinamide), second-line injectable drugs (amikacin, kanamycin, and capreomycin), and fluoroquinolones in combination with the second-line drugs. Drug-resistant TB has emerged due to factors that include improper treatment; poor quality, limited supply and cost of drugs; person-to-person transmission of resistant bacteria; and poor compliance. Drug resistance is now a major obstacle to effective global TB management and prevention^2^. In 2017, 30% of the 6.7 million new or TB relapse cases were reported to have resistance to rifampicin (the first-line drug of treatment) worldwide. Thus, the need of the hour is to develop novel, effective, and safer treatment strategies for the treatment of multidrug resistant (MDR) and extensively drug resistant (XDR) TB. However, the process of drug development, which includes a series of steps starting from pre-clinical studies to clinical trials, is time, cost, and labor extensive. This has resulted in a lack of new molecules that could be further developed and approved by the FDA for successful incorporation into anti-TB treatment strategies. Drug repurposing therefore emerges as a novel approach for drug development, as it merely involves discovering new targets for pre-existing drugs. Repurposing can be viewed as a highly effective, time-saving, and low risk drug development strategy, since the drug to be repurposed has already passed all the clinical trials^1^.

*M.tb* spreads via aerosols from infected individuals with active pulmonary disease. Transmission occurs when a person inhales droplet nuclei containing *M.tb* and the droplets reach the alveoli of the lungs. The bacteria are engulfed by alveolar macrophages into phagosomes. In principle, fusion of lysosomes and phagosomes leads to the elimination of foreign particles; however, *M.tb* has evolved mechanisms that prevent the fusion of phagosomes and lysosomes, thereby promoting its survival inside the alveolar macrophages^3^. *M.tb* possesses various signal transduction mechanisms that include eleven two-component systems, eleven eukaryotic-like Ser/Thr protein kinases (PknA-PknL)^4^, two protein tyrosine (Tyr) phosphatases (PtpA and PtpB) and a recently discovered protein tyrosine kinase (PtkA)^5,6^. These signaling molecules block the host defense pathway and allow the bacteria to adapt and survive within the host macrophages^7^. *M.tb* PtpA binds to the H subunit of the vacuolar H^+^-ATPase (V-ATPase) and dephosphorylates the host macrophage protein VPS33B, which prevents phagosome-lysosome fusion and phagosome acidification^8,9^. PtkA, which is encoded within the same operon as PtpA, mediates the phosphorylation of PtpA on Tyr128 and Tyr129 and enhances the activity of PtpA, which suggests a central role for PtkA in *M.tb* virulence^8,10^.

Structural studies of PtkA (30.6KDa; 291 amino acids (AA)) have shown that it contains two domains, an intrinsically disordered domain (IDD) and a kinase core domain (KCD). The IDD is the N-terminal domain, IDD_PtkA_ (80AA)^11^ and the KCD is the C-terminal domain KCD_PtkA_ (216 AA). The KCD_PtkA_ contains a catalytic loop (D^85^LD motif), lysine residues (Lys184, Lys217, and Lys270) for ATP binding, and an autophosphorylation site at Tyr262. The catalytic site of PtkA resides on the KCD, and its activity depends upon its interaction with the IDD. The IDD inhibits the activity of PtkA, as it masks the auto phosphorylation site on KCD_PtkA_. PtkA shows two conformational states-an open state conformation in which IDD is away from Tyr262 (auto phosphorylation site), making it accessible for phosphorylation and thereby increasing the activity of PtkA; and a closed state, in which IDD masks the Tyr262, thereby decreasing the activity of PtkA. Phosphorylation of IDD by Ser/Thr kinases (PknA) induces conformational changes in IDD, which move IDD away from the autophosphorylation site and promotes an open state of PtkA^12^.

The catalytic activity of KCD_PtkA_ alone is greater than the catalytic activity of KCD_PtkA_ measured along with IDD_PtkA_, which suggests an inhibitory role of IDD_PtkA_ for the catalytic activity of PtkA^12^. Here, we present a rigorous study in which we have performed Replica Exchange Molecular Dynamics (REMD) to investigate the structural conformers of the disordered domain IDD_PtkA._ Further we performed protein-protein docking of IDD_PtkA_-KCD_PtkA_ to analyse the impact of IDD on the catalytic activity of PtkA via study of IDD_PtkA_-KCD_PtkA_ interactions and through modification of the KCD region involved in catalysis. In the subsequent steps, molecular docking and simulations were performed with FDA-approved drugs against PtkA (IDD_PtkA_-KCD_PtkA_) to identify potential *M.tb* PtkA inhibitors.

## Results and Discussion

### IDD_PtkA_ modelling and validation

The structure of IDD of PtkA was modelled using the I-TASSER server^13^. The highly significant structural templates used in the modeling of the IDD protein domain through I-TASSER are listed in Table 1. As is evident from the good coverage and high Z score (>1 stands for a good alignment) in case of most of the templates, the generated threading alignment pointed towards a good and confident model. The quality of the modelled structures was validated through Protein Server Validation Suite (PSVS)^14^ and the highest quality model was selected on the basis of Ramachandran analysis of ψ/φ angle from PROCHECK. The plot showed a coverage of 61.7% of the residues in the most favored region, 26.7% of the residues in additionally allowed regions, and with a stretch of about 3.3% residues in disallowed regions. This indicated that, overall, 94% of the residues were in allowed regions, showing that the quality of the protein model was suitable for further study.

**Table 1:** Provides the highly significant structural templates for sequence alignment obtained from PDB library for modeling through I-TASSER.

PDB ID	Normalized Z-score
2nbiA	1.00
4ujiA	0.32
6edoA	0.93
2nbiA	0.68
4ijy	0.75
1i96R	0.70
3j9aA	1.74
1ztn	0.82
1wlpA	0.02
1ztn	0.60

### Conformational analysis of IDD_PtkA_

**Figure 1:**
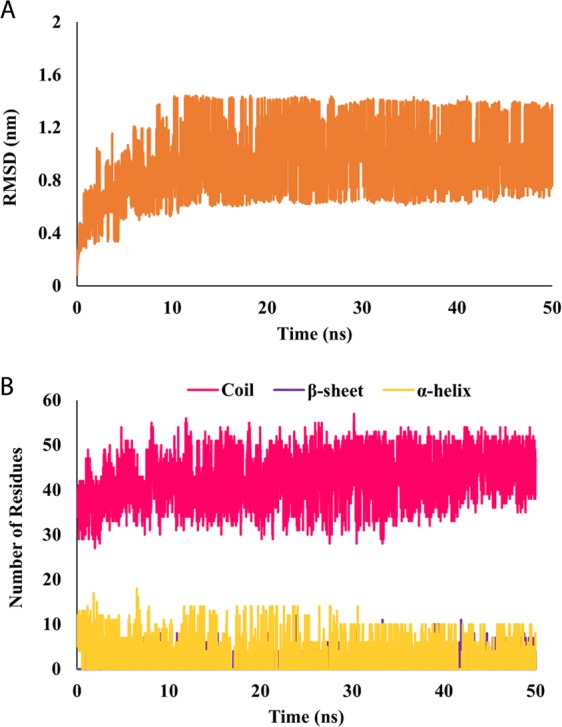
Depicts the plot of (**A**) RMSD and (**B**) secondary structure elements in IDDPtkA.

**Figure 2:**
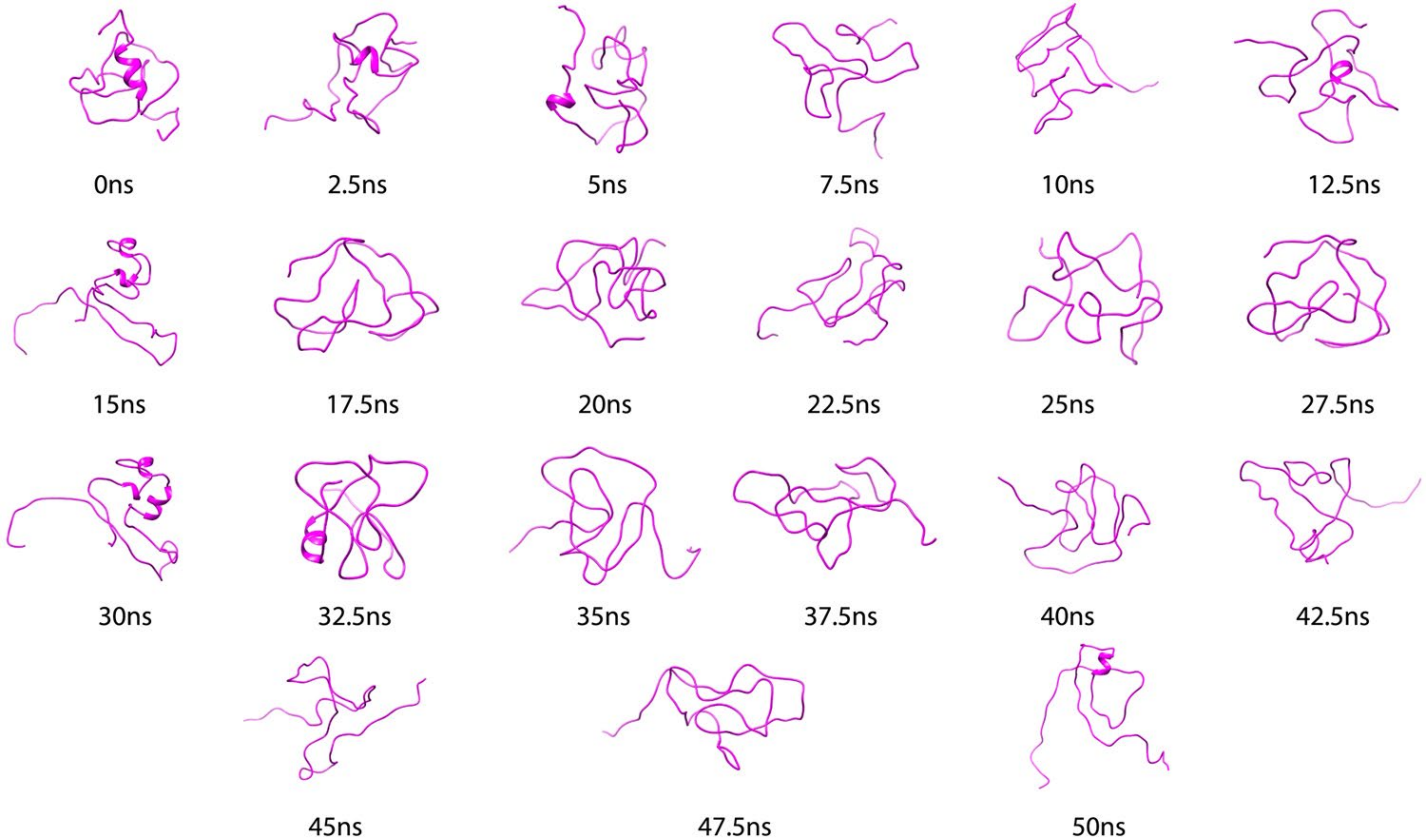
Snapshots of IDD taken at every 2.5 ns during 50 ns MD simulations.

The binding of IDD to KCD in PtkA has been shown to play an inhibitory role and to decrease the catalytic activity of KCD^12^. In a recent study, the conformation of IDD_PtkA_ has been characterized under varying biophysical conditions and phosphorylation using NMR-spectroscopy and it was confirmed that N-terminal domain of PtkA exists as an unstructured state^12^. REMD simulations was conducted on IDD_PtkA_ to examine the conformational behavior of the peptide at different temperatures. A total of 28 replicas were generated in the temperature range of 300–500 K, and REMD analysis was conducted for a replica at 300 K. Conformational analysis of IDD provided insight into the unstable and disordered structure of IDD. Since IDD is an intrinsically disordered protein, the root mean square deviation (RMSD) plot exhibited large fluctuations. However the fluctuations lied within a range of 0.7–1.3 nm throughout the simulations, thus indicating the characteristic flexible nature of IDD as well as convergence of the simulations. Since the protein was stable throughout the simulations period; the protein was considered for further analysis. Figure 1 depicts the plot of (A) RMSD and (B) secondary structure elements in IDD_PtkA._ The secondary structure analysis of IDD_PtkA_ revealed that throughout the entire simulations period (50 ns), IDD acquired multiple conformations; these were mostly random coils, along with short stretches of unstable α-Helices/β-Sheets. Snapshots of IDD taken every 2.5 ns (Fig. 2) during the 50 ns MD simulations evidently speak about this conclusion that the protein tyrosine kinase A (PtkA) has an 80-residue intrinsically disordered N-terminal region. The coil percentage in IDD was 54%, whereas the α-Helices and β-Sheets were present at a mere 3% and 1%, respectively. This indicated that no persistent secondary structure elements were present in IDD_PtkA_.

### Inhibitory activity of IDD_PtkA_ on KCD_PtkA_

**Figure 3:**
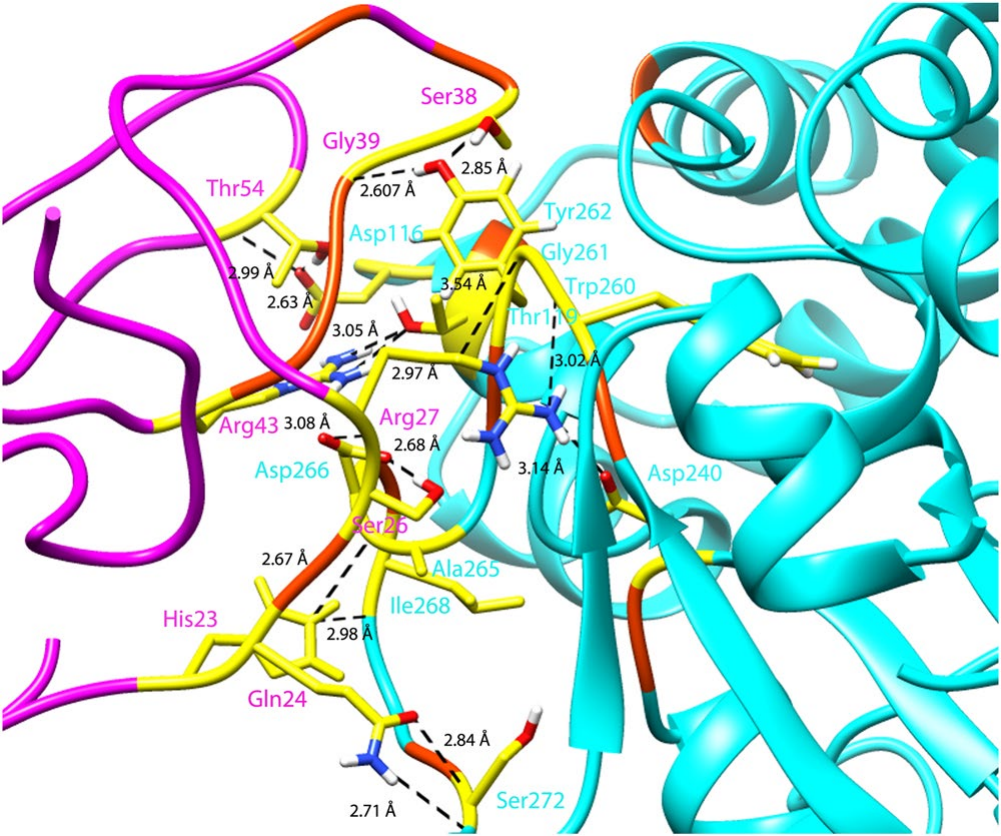
Illustrate interaction pattern between IDD and KCD domains of PtkA; IDD_PtkA_ (magenta), KCD_PtkA_ (cyan), hydrogen bonds (yellow), hydrophobic residues (orange).

Since the inhibitory effect of IDD on KCD has already been verified^12^, protein-protein docking and MD simulations of IDD_PtkA_-KCD_PtkA_ were conducted to explore the hydrophobic and hydrogen bond interaction patterns between the two domains. The top-scoring cluster having a HADDOCK score of −58.6+/− 9.0 kcal/mol and Z-score equivalent to −1.2 was considered for MD simulations. The top cluster is considered to represent the most reliable cluster on the basis of Z-score which indicates the magnitude of standard deviations between the given cluster and the average of all other clusters (the more negative the better). RMSD, root mean square fluctuation (RMSF), radius of gyration (Rg), and solvent accessible surface area (SASA) analysis of PtkA in comparison to PtkA-drug complexes during MD simulations is described later in the manuscript. The residues His23, Gln24, Ser26, Arg27, Ser38, Gly39, Arg43, and Thr54 of IDD formed hydrogen bonds with the KCD residues Asp116, Thr119, Asp240, Trp260, Gly261, Tyr262, Asp266, Ile268, and Ser272. Among these were residues that have been reported to form interactions between IDD and KCD that include Glu114 and Trp260; the other active site and surrounding residues formed hydrophobic interactions. This pointed out that IDD is important for regulating the catalytic activity KCD and thus reducing the efficiency of PtkA. Figure 3 illustrates the hydrogen bonding pattern and the hydrophobic interactions between IDD and KCD.

### Identification of lead compounds against PtkA

In order to identify drugs that can be repurposed as agents against *M.tb* PtkA, we performed molecular docking studies using the FDA-approved drugs as ligands. Initially, high throughput virtual screening (HTVS) docking was performed, followed by extra precision (XP) docking using the top scorers of HTVS (having docking score <−5.0). The two top scoring drugs, esculin (DrugBank ID: 13155) and inosine pranobex (DrugBank ID: 13156), had Glide scores equivalent to −8.44 kcal/mol and −8.82 kcal/mol, respectively. Esculin had a Glide energy of −70.99 kcal/mol and inosine pranobex had a Glide energy of −67.97 kcal/mol. Esculin formed one hydrogen bond each with Val122 and Ser209, while inosine pranobex formed four hydrogen bonds with residues Ile21, Lys184, and Ser209.

We also conducted docking of both drugs with KCD_PtkA_ without IDD_PtkA_ to clarify the role of IDD_PtkA_ in preventing the catalytic activity of PtkA. The docking score of KCD_PtkA_-inosine pranobex was −6.11 kcal/mol and that of KCD_PtkA_-esculin was −4.59 kcal/mol. The lower docking scores in the case of KCD without IDD suggested a strong binding of drugs in the presence of IDD, indicating a significant role of IDD in the structure of KCD. The docking scores, hydrogen bonding, and hydrophobic interactions of PtkA (IDD-KCD) with esculin and inosine pranobex and with PtkA (KCD) are provided in Table 2.

**Table 2:** Provides the docking scores, pre-MD hydrogen bonding and hydrophobic interactions of PtkA (IDD-KCD) with the drugs, esculin and inosine pranobex, and PtkA (KCD).

Drugs	PtkA (IDD-KCD)			Docking scores (kcal/mol)	PtkA (KCD)	
	Docking scores (kcal/mol)	H-Bonds	Hydrophobic Residues		H-Bonds	Hydrophobic Residues
Inosine Pranobex	−8.82	Ser209	Val122	−6.11	Ile121	Asp85
		Ile121	Gly123		Gly123	Asp87
		Lys184	Pro124		Pro124	Ile97
			Pro125		Met126	Val122
			Met126			Pro125
			His127			Thr182
			Asp211			Lys184
			His243			Asp240
Esculin	−8.44	Ser209	Asp87	−4.59	Asp87	Asp85
		Val122	Gly88		Asp240	Gly88
			Gly123		Asp244	Pro125
			Thr182			Met126
			Lys184			Thr182
			Thr210			Ser183
			Asp211			Lys184
			Arg241			Gly239
			Ser242			Arg241
			His243			His243
			Arg264			
			Ile268			

### Elucidation of the mechanism of binding of drugs to PtkA

**Figure 4:**
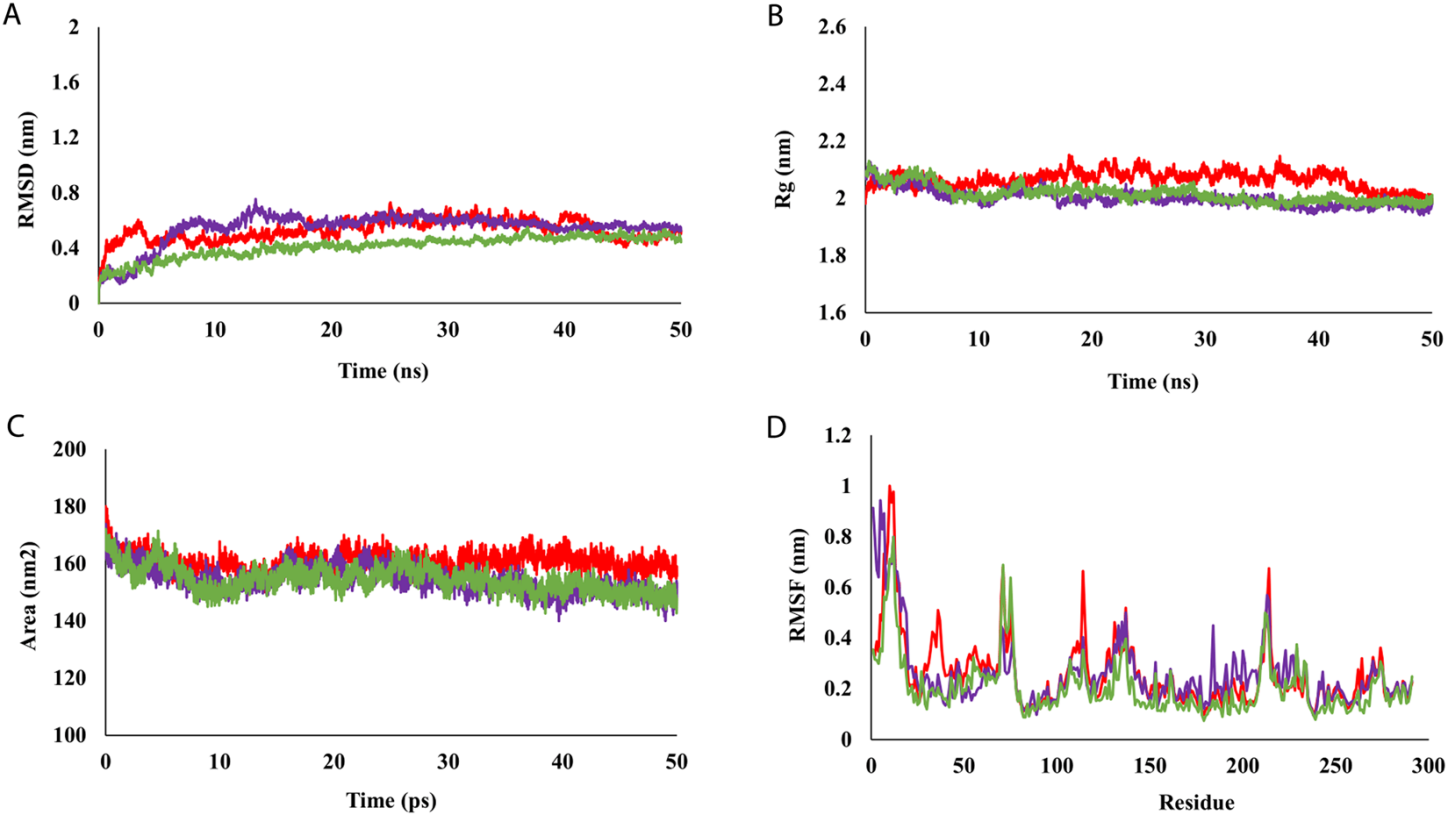
Represents the plots obtained during MD simulations of unbound and drug-bounded PtkA (**A**) RMSD (**B**) Rg (**C**) SASA (**D**) RMSF; PtkA (red), PtkA-esculin (purple) and PtkA-inosine pranobex (green).

**Figure 5:**
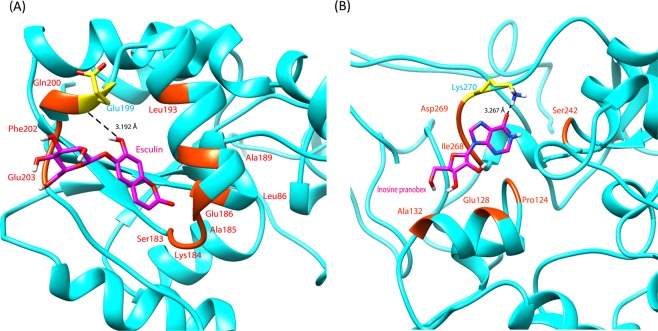
Hydrogen bonding pattern of the drugs (magenta), esculin and inosine pranobex, with PtkA (cyan).

In order to gain insights into the thermodynamic behavior and to further examine the mechanism of drug binding and its impact on PtkA, the docked protein-ligand complexes were subjected to 50 ns MD simulations. The RMSD plots of PtkA, PtkA-esculin, and PtkA-inosine pranobex showed that the protein and protein-drug complexes were stable during the simulations and were therefore suitable for further analysis (Fig. 4(A)). The Rg graph showed a decrease in the overall Rg value of the protein structure docked with drugs, indicating that PtkA in complex with drugs was in a compactly packed state and had stable folding (Fig. 4(B)). The SASA calculation suggested that the exposure of the protein surface to the solvent and the changes in SASA could lead to conformational changes. Figure 4(C) shows the variations in SASA for the protein and protein-drug complexes with respect to time. The SASA values for the drug-bound PtkA were reduced when compared with the case of the unbound PtkA. The increased SASA values indicate a partial unfolding of the protein structure upon exposure to solvent. However, the binding of the drugs to PtkA resulted in decreased SASA values, which denote the comparatively shrunken nature of the protein. Figure 4(D) shows a clear difference in the fluctuation scores in the residues of the protein-drug complexes as compared to the unbound protein. The high RMSF values of PtkA indicated a larger degree of flexibility and instability in the protein, whereas the low RMSF values in the cases of the PtkA-esculin and PtkA-inosine pranobex complexes showed restricted movement of the residues and a rigid structure for PtkA in the presence of the drugs. The binding pattern of esculin and inosine pranobex with PtkA are shown in Fig. 5. The average values of RMSD, Rg, SASA, and RMSF (active site residues) during the simulations for the protein (PtkA) and protein-drug complexes (PtkA-esculin and PtkA-inosine pranobex) are summarized in Table 3. The values indicate that inosine pranobex binding had an overall more stabilizing effect on PtkA when compared to esculin.

**Table 3:** Signifies the average values of RMSD, Rg, SASA and RMSF (active site residues) during the simulations time period for protein (PtkA) and protein-drug complexes (PtkA-esculin and PtkA-inosine pranobex).

	PtkA	PtkA-inosine pranobex	PtkA-esculin
Rg (nm)	2.07	2.01	2.00
SASA (nm)	155.63	147.80	153.26
**RMSF (nm) (Active site residues)**			
Asp85	0.11	0.09	0.12
Leu86	0.13	0.12	0.15
Asp87	0.13	0.13	0.19
Lys184	0.18	0.15	0.44
Lys217	0.21	0.27	0.26
Lys270	0.32	0.24	0.31
Tyr262	0.27	0.19	0.13
Trace value of covariance (nm^2^)	74.10	47.43	66.96

### Principal component analysis

**Figure 6:**
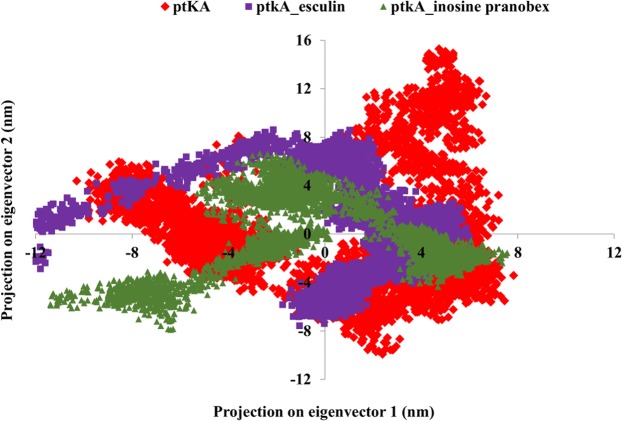
Projection of motion of protein atoms of (**A**) PtkA, (**B**) PtkA-esculin and (**C**) PtkA-inosine pranobex on PC1 and PC2.

The collective movement of the atoms in the unbound and ligand bound PtkA was analyzed using the MD trajectories projected on principal components (PC1 and PC2) to obtain a better understanding of the conformational changes in PtkA. The Eigen vectors revealed the general direction of motion of the atoms, while the Eigen values denoted the atomic influence in movement. A large distribution of dots indicated greater variance in accordance with more conformational changes in the PtkA protein. The PtkA trajectories showed higher space magnitudes and covered a wider conformational space, supporting accelerated collective motions in the unbound protein when compared with the protein-drug docked complexes (Fig. 6).

**Figure 7:**
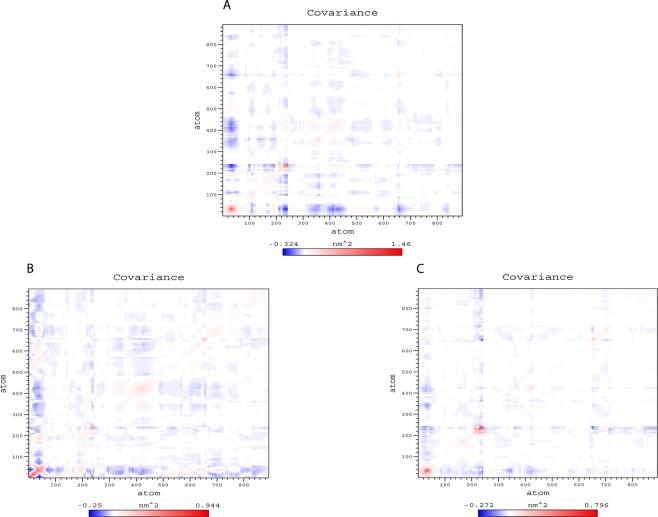
Shows the diagonalized covariance matrix of (**A**) PtkA (**B**) PtkA-esculin and (**C**) PtkA-inosine pranobex.

The flexibility of the protein was analyzed to a larger extent by calculating the trace value for diagonalized covariance matrix, which is a matrix of Eigen vectors and diagonal Eigen values. Figure 7 shows the diagonalized covariance matrix of PtkA, PtkA-esculin, and PtkA-inosine pranobex. The trace values, which are the sums of the Eigen values, were 74.10, 66.96, and 47.43 nm^2^ for PtkA, PtkA-esculin, and PtkA-inosine pranobex, respectively. Therefore, unbound PtkA appeared to cover a larger conformational space due to its greater flexibility when compared with the protein-drug complexes.

### Free energy analysis

**Figure 8:**
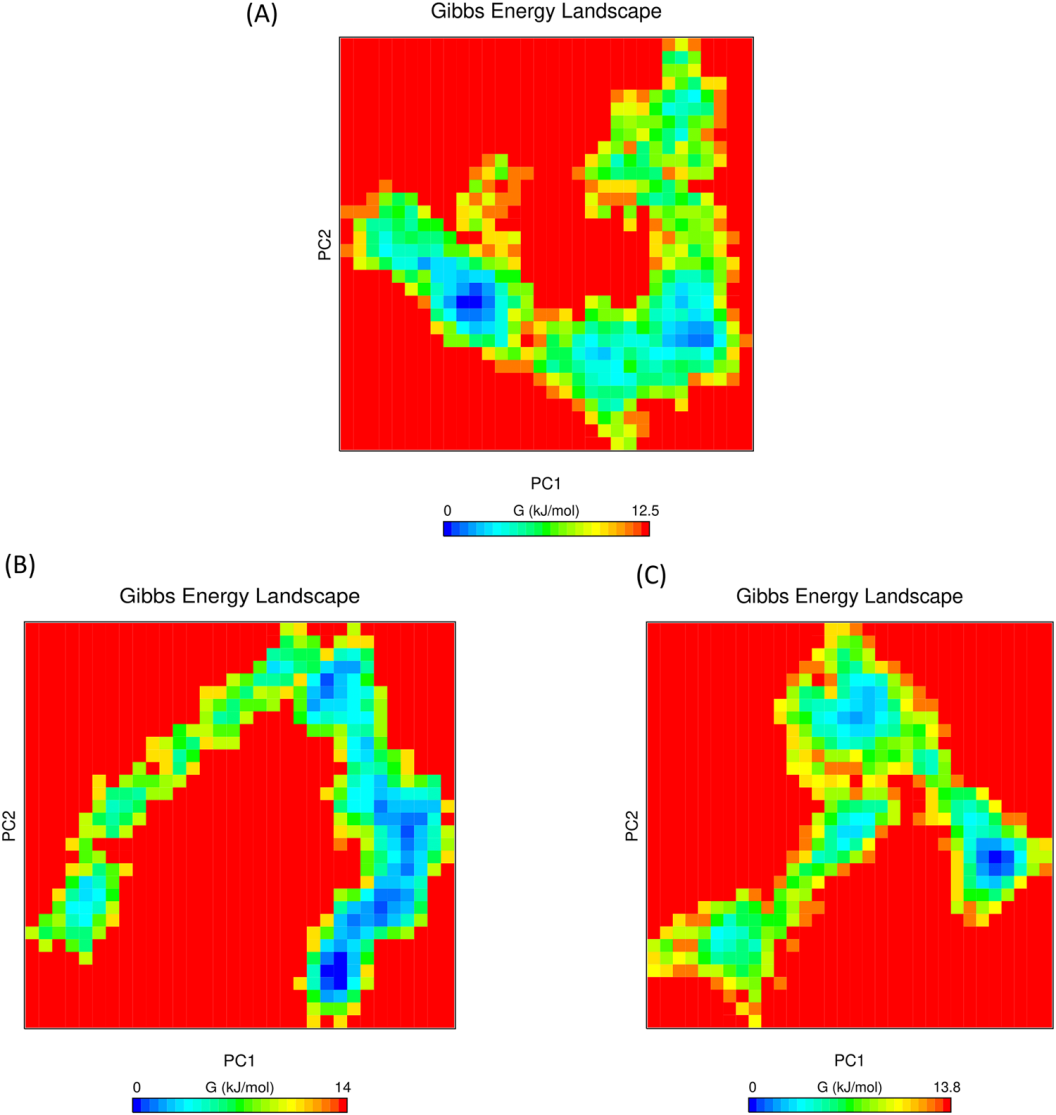
Shows the Gibbs free energy landscape plot of (**A**) PtkA and (**B**) PtkA-esculin and (**C**) PtkA-inosine pranobex.

Gibbs free energy landscape (FEL) plot for unbound protein (PtkA) and protein-drug complexes (PtkA-esculin and PtkA-inosine pranobex) was generated using PC1 and PC2 coordinates. The ΔG values for PtkA, PtkA-inosine pranobex and PtkA-esculin were 12.5, 13.8 and 14 kJ/mol respectively. The blue, cyan and green regions in the plot signify low energy state with highly stable protein conformation while the red region denotes high energy conformation. A smaller and more concentrated blue minimal energy area in PtkA-inosine pranobex and PtkA-esculin indicates a highly stable complex as compared to PtkA alone (Fig. 8).

### Secondary structure analysis

The secondary structure elements included rigid (α-Helices and β-Sheets) conformations and flexible (coils and turns) conformations. The unbound PtkA was conquered by coil (28%) which decreased to 26% and 27% in case of PtkA-inosine pranobex and PtkA-esculin respectively (Table 4). An increase in β-Sheet and turn was observed in case of PtkA-inosine pranobex complex favouring stability in the presence of inosine pranobex. Furthermore Table 5 provides the secondary structure elements adopted by the active site residues of unbound PtkA and PtkA-drug complexes. An increase in rigid structural elements at active site residues was observed in case of PtkA-inosine pranobex. Most of the active site residues retained their conformation in case of PtkA-esculin except Lys270 which changed its conformation from coil to turn favouring a stable complex.

**Table 4:** Overall percentage of secondary structure elements in unbound PtkA and PtkA-drug complexes.

Drug/protein	Coil %	α-Helix %	β-Sheet %	Turn %	3_10_ Helix %	β-Bridge %
PtkA (IDD-KCD)	28	3	10	12	2	2
PtkA-inosine pranobex	26	3	11	13	2	1
PtkA-esculin	27	3	9	12	2	2

**Table 5:** Comparative secondary structural elements at active site residues of PtkA in unbound and drug-bound state.

Residue No.	PtkA (IDD-KCD)	PtkA-Inosine Pranobex	PtkA-Esculin
Asp85	Coil	Coil	Coil
Leu86	π-Helix	α-Helix	π-Helix
Asp87	π-Helix	α-Helix	π-Helix
Tyr146	α-Helix	α-Helix	α-Helix
Tyr150	α-Helix	α-Helix	α-Helix
Lys184	Turn	Coil	Turn
Lys217	α-Helix	α-Helix	α-Helix
Tyr262	Coil	Coil	Coil
Lys 270	Coil	Turn	Turn

## Conclusion

Protein phosphorylation and dephosphorylation at tyrosine residues have a significant role in the survival of *M.tb*. PtpA and PtkA are the two protein tyrosine phosphatases secreted by *M.tb*. PtpA is an essential protein for *M.tb* pathogenesis, as it prevents phagosome acidification and plays a significant role in virulence. The structure of PtpA shows a high similarity to that of human protein phosphatase; thus, it cannot be considered a suitable target for drug development. The phosphatase activity of *M.tb* PtpA is regulated by PtkA, which is composed of a disordered IDD domain and a rigid catalytic KCD domain. PtkA phosphorylates Tyr128 and Tyr129 of PtpA, thereby enhancing its activity, and it has also recently been reported to increase the growth of *M.tb* in macrophages. Thus, *M.tb* PtkA can be a suitable potential target for drug development against TB. The structure analysis of PtkA showed that the disordered domain, IDD, modulates the arrangement of the helices involved in the catalytic activity of KCD, thereby showing a significant role for IDD. We examined the molecular mechanism behind the role of PtkA in regulation of phosphorylation by *M.tb*. PtpA by performing an extensive computational study on PtkA that included modelling, REMD, MD, and molecular docking. Since the structure for IDD was not available, it was modelled and validated for quality assessment and subjected to REMD. Notably, IDD was largely composed of random coils that transiently bind to the substrate binding site of KCD. The docking and MD of IDD and KCD domain, performed to look at the inhibitory effect of IDD on KCD, revealed strong bonding between IDD and KCD.

Potential inhibitors against PtkA (IDD-KCD) were identified using molecular docking and resulted in two top scoring drugs, esculin and inosine pranobex. Various analyses, RMSD, RMSF, Rg, SASA, and PCA, done using MD trajectories indicated that IDD, in the presence of the drugs, had a greater inhibitory effect on the catalytic activity of KCD. The results also revealed that PtkA was highly stable in the drug-bound form, when compared to unbounded PtkA. This study proposes the repurposing of two FDA approved drugs, esculin and inosine pranobex, for use as agents against PtkA and consequent inhibition of the growth of *M.tb*.

## Methodology

### Homology modeling of IDD_PtkA_ and quality assessment

The sequence of IDD_PtkA_ (N-terminus) of *M.tb* was retrieved from UniProt-KB database [UniProt ID: P9WPI9]^15^. Since the crystal structure of IDD was not available, the structure was modeled on the basis of sequence homology through I-TASSER server^13^. I-TASSER has regularly been the top ranked method in CASP (Critical Assessment of Protein Structure Prediction) method (Moult *et al*. 2014). I-TASSER initiates modelling by identifying template proteins from PDB library. The models are then constructed by continuous assembling of the aligned templates and initiating *ab initio* folding for the unaligned regions based on replica exchange Monte Carlo simulations. These simulations generate an ensemble of conformations which are further clustered on the basis of free energy. Further lowest energy structures are subjected to refinement resulting in final structural model^16^. Structure quality assessment was performed by PSVS^14^ through quality scores and constraint analysis programs. The modelled protein structure was subjected to 50 ns REMD simulations and the stable conformation was used for further analysis.

### Replica exchange molecular dynamics (REMD) simulations

REMD is a molecular simulations method that enhances the sampling efficiency. A set of replicas in a canonical ensemble are simulated at different temperatures, and at particular intervals, the replicas are swapped with a transition probability such that each independent system maintains a canonical ensemble. These exchanges allow the molecule to jump out of the minima and sample efficiently on the conformational space^17^. It has been reported that the 80-residue N-terminal domain of PtkA protein is an intrinsically disordered region which lacks a stable structure and goes through conformational exchange on changing the environmental settings such as temperature, chemical denaturant and phosphorylation^12^. In the present study, to explore the conformations of IDD_PtkA_, REMD was performed at varying temperatures ranging from 300–500 K.

REMD simulations of IDD were performed using GROMACS version 5.0^18^ with GROMOS 43a1 force field. The peptide was provided with periodic boundary conditions (PBC) and placed in a cubic box filled with a single point charge (SPC)^19^ water model containing 710 water molecules. Na and Cl ions were added to neutralize the overall charge on the system. A total of 28 replicas were swapped in a temperature range of 300K-500K (300.00, 306.23, 312.56, 318.98, 325.51, 332.14, 338.89, 345.75, 352.71, 359.78, 366.97, 374.25, 381.69, 389.24, 396.92, 404.72, 412.66, 420.72, 428.91, 437.23, 445.70, 454.30, 463.05, 471.95, 480.98, 490.14, 499.48, and 508.97) with an acceptance exchange probability of 0.20. The temperature was predicted according to the method described by Patriksson and van der Spoel^20^. The REMD simulations was carried out for 50 ns. Further RMSD analysis was performed to study the convergence of simulations.

### Docking and molecular dynamics simulations study of IDD_PtkA_-KCD_PtkA_

The X-ray crystal structure of KCD was obtained from a protein data bank (PDB ID: 6F2X). The IDD and KCD of PtkA were docked using the HADDOCK^21^ web server. The server performs data-driven flexible docking, in which it integrates the experimental data (X-ray and NMR) as restraints and uses these to implement docking, in addition to a combination of energetics and shape complementarity. An AIR (ambiguous interaction restraints) file which consist of active and passive residues at the interaction interface for each molecule based on NMR data, was supplied to the server. Active residues are the residues that are experimentally determined to be involved in the interaction between the two molecules. For the current study the active residues involved in the interaction between IDD and KCD has been obtained from experimental data^12^. The active site of IDD included residues Ala10, Ser41, and Cys61, and the KCD active site consisted the residues Glu114, Gly134, Asp162, Thr188, Ile192, Ile205, Gly212, Leu231, Met237, Trp260, and Ile282. The docked complex (IDD_PtkA_-KCD_PtkA_) was simulated under the dynamic behavior of molecular systems as a function of time using GROMACS version 5.0 with the GROMOS 43a1 force field. The complex was provided with PBC and placed in a cubic box filled with the SPC water model. The energy of the system was minimized until the system attained the maximum force <1000.0 kJ/mol/nm to ensure no steric clashes on the system. The system was first equilibrated under an isothermal-isochoric ensemble (NVT, constant Number of particles, volume, and temperature) using a Berendsen thermostat (a v-rescale) applied for temperature coupling for a total of 50000 steps. This was followed by equilibration under an isothermal-isobaric ensemble (NPT, constant number of particles, pressure, and temperature) using Parrinello-Rahman applied for pressure coupling, again for 50000 steps. The two phases of equilibration stabilized the system at 300 K and 1 bar pressure, and the system was subjected to MD simulations for 50 ns. The LINCS constraints algorithm was applied to fix the lengths of the peptide bonds and angles^22^. The MD trajectory files were analyzed using various inbuilt scripts of GROMACS.

### Molecular docking of PtkA (IDD-KCD) and MD simulations

The complete protein structure of *M.tb* PtkA was then used for the molecular docking study to identify potential inhibitors of PtkA. The docking studies were conducted using the Schrodinger suite. Prior to the docking, the target protein was pre-processed by adding missing hydrogen atoms, correcting bond orders, and capping protein termini using the Protein Preparation Wizard^23^. A library of 2355 FDA approved drugs was obtained from the DrugBank^24^ database and LigPrep^23^ was used to prepare the chemical structures of the drugs. This resulted in energy-minimized, chemically broad, and structurally diverse 3D structures of the drug molecules. The Receptor grid generation panel was used to generate a grid around the active residues (Asp85, Leu86, Asp87, Tyr146, Tyr150, Lys184, Lys217, Tyr262 and Lys270) of KCD. The docking was performed in two steps: HTVS docking and XP, using the Glide module of Schrodinger^25^. The large chemical library was first screened using the HTVS approach. A rapid screening of the ligands was executed, followed by the XP method, which implements a rigorous screening of the top-scoring HTVS ligands and results in compounds having the best binding mode to the receptor. The top-scoring protein-ligand complexes were subjected to 50 ns MD simulations using GROMACS 5.0. The webserver PROGRG^26^ was used to generate GROMACS topologies for the ligands.

### Molecular dynamics simulations analysis

The MD simulations runs performed in the present study were analyzed using various GROMACS tools. RMSD was calculated using g_rmsd to study the convergence of the simulations. Rg of the protein, which is a measure of the protein folding and compactness, was calculated using g_ gyrate^27^. RMSF for deviation in the position of atoms was calculated using g_rmsf. The SASA for the protein, which gives an idea of the area of the residues exposed to the surface, was calculated using g_sasa. We also performed essential dynamics or PCA, which gives a view of the large-scale combined motion of atoms, thereby revealing the behavior of the protein structure underlying the atomic variations. The initial step in PCA is to construct a covariance matrix (g_covar), which captures the linear relationship of atomic fluctuations for individual atomic pairs. This is followed by diagonalization of this matrix, which results in a matrix of eigenvectors and eigenvalues. Eigenvectors determine the movement of atoms and eigenvalues yielded by diagonalization represent the extent that an atom participates in motion. The Eigen vectors were analyzed using the g_anaeig tool. Further FEL was calculated using g_sham module and the secondary structures of PtkA and PtkA-drug complexes were analysed using gmx_dssp. LigPlot^28^ was used to explore the hydrogen and hydrophobic interactions, and Chimera^29^ was used to create the images depicting the interactions.
